# Relevance of Smoking Habit in Severe Asthma Patients: Evidence from the Severe Asthma Network in Italy (SANI) Registry

**DOI:** 10.3390/jcm11247465

**Published:** 2022-12-16

**Authors:** Marco Caminati, Gabriella Guarnieri, Pierluigi Paggiaro, Andrea Vianello, Ernesto Crisafulli, Rachele Vaia, Gianenrico Senna

**Affiliations:** 1 Department of Medicine, University of Verona, 37134 Verona, Italy; 2 Department of Cardiac Thoracic Vascular Sciences and Public Health, University of Padova, 35122 Padua, Italy; 3 Department of Surgery, Medicine, Molecular Biology and Critical Care, University of Pisa, 56126 Pisa, Italy; 4 Respiratory Medicine Unit, Department of Medicine, University of Verona and Verona University Hospital, 37134 Verona, Italy; 5 Allergy Unit and Asthma Center, Verona University Hospital, 37134 Verona, Italy

**Keywords:** severe asthma, cigarette smoking, Severe Asthma Network in Italy, eosinophils, lung function

## Abstract

Smoking habit is still fairly common among asthmatics. So far, the impact of smoke on severe asthma burden has not been specifically investigated. We aimed to estimate the frequency of smoking habit among severe asthma patients, their clinical features, and the impact of smoke on asthma outcomes. The Severe Asthma Network in Italy (SANI) registry was analyzed. Demographic, clinical, and functional features of smokers, never and former smokers were compared. Data from 1194 patients were explored. Smokers were younger, with a lower asthma onset age. Atopy, BMI and respiratory/systemic comorbidities were equally distributed. In former smokers pre- and post-FEV1/FVC was significantly lower; no other significant differences were detected. Similar findings were confirmed when stratifying the former smokers by pack-years and length of smoking cessation. Among former smokers, lymphocytes and neutrophils were higher in the <15 years of smoking cessation group. Blood eosinophils were comparable in never and former smokers. When clustering the population by blood eosinophils, no significant differences in pulmonary function and exacerbations were observed. Our data suggest that a personal smoking history has a relatively low impact on disease burden. It remarks the importance of smoking cessation as a main intervention, particularly in severe asthma.

## 1. Introduction

It is well known that cigarette smoking exerts a number of negative effects, especially in patients affected by chronic respiratory diseases, including bronchial asthma. Through several mechanisms smoke exposure leads to worsening of asthma symptoms, lung function decline, immune impairment and reduced response to oral and inhaled steroids [1]. Nevertheless, according to several reports, smoke is still fairly common among asthmatics; around one out of four subjects with asthma is a current smoker [1,2]. So far, the impact of smoke on severe asthma burden has not been specifically investigated.

Our study aimed to estimate the frequency of smoking habit among severe asthma patients, their clinical features, and the impact of smoke exposure to severe asthma outcomes.

## 2. Materials and Methods

The Severe Asthma Network in Italy (SANI) registry was analyzed. SANI collects data from patients affected by severe asthma, according to the ERS/ATS definition [3] from Italian referral centers with particular expertise in the diagnosis and the management of severe asthma. The registry protocol is reported in detail elsewhere [4]. According to smoking habits, three subgroups were identified: smokers, former smokers, and non-smokers. The first subgroup included current smokers. The second subgroup included ex-smokers for at least the past 6 months with a smoking history of at least 5 pack-years. The third subgroup included nonsmokers for at least the past 12 months with a smoking history of ≤5 pack-years. A preliminary Shapiro–Wilk test was performed. Data are reported as percentages for categorical variables and as median [interquartile range] for continuous variables due to the non-normal distribution. Categorical variables were compared by the χ2 test or the Fisher exact test, while continuous variables were assessed by the non-parametric Mann–Whitney test. All analyses were performed using IBM SPSS, version 17.0 (IBM Corp., Armonk, NY, USA), with *p*-values of <0.05 considered statistically significant.

## 3. Results

Baseline data from 1248 patients have been explored. According to smoking habits, three subgroups have been identified: smokers (*N* = 45), former smokers (*N* = 239), and non-smokers (*N* = 910). Their demographic data are shown in Table 1.

Smokers were significantly younger (mean age 49 years old vs. 59 for former smokers and 56 for non-smokers), and a lower asthma onset age was observed in the same subgroup (31.5 years old vs. 39 for former smokers vs. 34 for non-smokers). Regarding gender, a higher proportion of males was described in the former smokers group (48%) vs. no smokers (36%) and current smokers (38%). On the opposite, females were more prevalent in the no smokers cluster. No significant differences among subgroups could be described in terms of the presence of atopy, BMI, and respiratory or systemic comorbidities prevalence (namely: rhinitis, chronic rhinosinusitis, nasal polyposis, bronchiectasis, GERD, atopic dermatitis, urticaria, psoriasis, chronic cardiovascular disease, depression, anxiety, diabetes, osteoporosis). Due to their low number, the current smokers were excluded from further statistical analyses. Table 2 summarizes functional, clinical, and hematological data.

**Figure 1:**
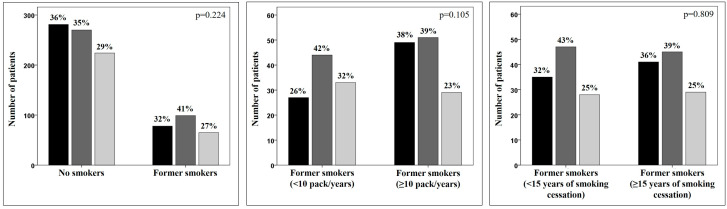
Clinical assessment according to the GINA recommendations definition of control. Black, gray and light gray bars represent patients not-controlled, partially controlled, and controlled, respectively.

In the former smokers subgroup, the pre- and post-FEV_1_/FVC was significantly lower; no other significant differences could be described regarding the other functional variables and clinical outcomes including exacerbations, emergency department admissions, and hospitalizations in the previous year. Asthma control test (ACT) was as well comparable in never smokers and former smokers. The same findings were confirmed when stratifying the former smokers by pack-years and length of smoking cessation. When considering the overall asthma control in terms of clinical assessment according to GINA recommendations (Figure 1), the proportion of not-controlled, partially controlled, and controlled patients is comparable in never and former smokers; furthermore, the number of pack-years within the former smokers subgroup is not relevant.

The lab parameters analyses showed that among former smokers, a significantly higher level of lymphocytes and neutrophils was present in the <15 years of smoking cessation group in comparison with the ≥15 years from smoking cessation one. Blood eosinophilic count showed no statistically significant differences between never and former smokers. However, when clustering the overall study population by blood eosinophils, we observed that FeNO was significantly higher in patients with blood eosinophils >300/mmc, in which we observed also worse self-reported outcomes in terms of ACT and ACQ questionnaires results. Nevertheless, we did not find statistically significant differences in pulmonary function, evaluated through spirometry, nor in exacerbation indexes between patients with higher and lower eosinophilic counts (Table 3).

## 4. Discussion

The presented findings from SANI database analysis point out a few differences between never and former smokers affected by severe asthma. In fact, except for a significantly lower pre- and post-FEV1/FVC in former smokers, the other analyzed functional and clinical parameters were comparable in the two groups.

These findings are quite surprising, as they suggest an impact of smoking habit on asthma control and history lower than expected, particularly when considering severe asthmatics.

Thomson and coauthors, by reviewing the British Thoracic Society Severe Asthma Registry, also reported some similarities shared by former and never smokers in terms of inflammation pattern (sputum eosinophils and FeNO) and asthma clinical control; on the opposite, current smokers were characterized by poorer asthma control, more unscheduled visits and major exacerbations requiring courses of oral steroids [5]. Similar results are described within The Belgian Severe Asthma Registry, reporting a much higher proportion of current smokers when compared to the SANI database (12% vs. 3.5%) [6].

As previously stated, due to the subgroup’s small size, the current smoker population has been excluded by our analysis, which limits the strength of evidence for the effects of current smoking status on clinical outcomes and may contribute to explaining our findings. However, when stratifying by pack-years and length of smoking cessation, the results remain substantially unchanged.

Differently from the above-mentioned analysis from the Belgian British and Italian severe asthma registries, Shaw and co-authors grouped together current and former smokers when exploring the European U-BIOPRED adult severe asthma cohort groups by smoking history [7]. In both groups, nonsmoking and the smoking/ex-smoking severe asthma patients, a similar oral corticosteroid intake and airflow obstruction was detected, whilst the smoking/ex-smoking group presented a slightly lower level of FeNO, as expected. Of note, a significant correlation between AQLQ scores and the number of pack-years was described, supporting the contribution of smoking habit to the disease burden and impact on quality of life.

On a clinical ground, few studies have specifically addressed as the primary outcome the impact of smoking in severe asthmatics. Konno and co-authors [8] identified two different inflammation patterns in severe asthmatic smokers; the first, comparable with our study population, was characterized by younger age higher eosinophil count, elevated total serum IgE and FeNO, and upper airways involvement. In the second cluster, patients were older and showed a lower eosinophilic count and total IgE dosage. When considering functional impairment patients in both the subgroups, all of them active smokers, were affected by more severe disease in comparison to our population, including former smokers only. It can be hypothesized that smoking status, current or former, makes a substantial difference in terms of asthma control and functional parameters.

Data investigating the relationship between eosinophilic inflammation and smoke may provide a key for the interpretation of our results. In fact, it has been demonstrated that blood eosinophil count is reduced in smokers with asthma when matched with a comparable never-smokers population [9], whereas it is increased in non-asthmatic smokers [10]. Unexpectedly, in our population, blood eosinophilic count did not show statistically significant differences between never and former smokers, even if it showed an increasing trend in former smokers. However, similar results were described by Lemiere et al. who compared active-smoker and non-smoker subjects affected by uncontrolled moderate-to-severe asthma. The authors reported similar clinical characteristics in the two sub-groups as well as comparable eosinophil count values. Of note, a higher proportion of non-smokers presented a sputum eosinophil count > 10% whilst asthmatic smokers had lower levels of FeNO. Anyway, the authors suggested a marginal impact of smoking on airway eosinophilic inflammation [11].

According to recent experimental data, the analyses of microRNA-218-5p in bronchial brushings of asthma patients have suggested that its expression within the cigarette smoking-induced inflammation cascade may play a kind of protective role. In fact, it targets CTNND2, a novel catenin, and suppresses chemokine CCL26 expression, both up-regulated in eosinophilic airway inflammation models [12]. This effect should be considered in light of the known long-term negative effects of smoking on respiratory chronic diseases.

## 5. Conclusions

Taken together, our data and the published evidence suggest that when comparing former smokers to never smokers, regardless of pack-years and length of smoking cessation, a personal smoking history has a relatively low impact on severe asthma burden, in terms of clinical outcomes and comorbidities. It remarks the importance of smoking cessation as a main intervention, particularly in severe asthma.

## Figures and Tables

**Table 1:** Demographic data and comorbidities of severe asthma study population.

	No Smokers	Former Smokers	Current Smokers	*p*-Value
	(*N* = 910)	(*N* = 293)	(*N* = 45)	
Age, y	56 [16]	59 [17] *	49 [25] ** §§	**<0.001**
Age of asthma onset, y	34 [26]	39 [25] *	31.5 [33] §	**0.004**
Gender, females, *n* (%)	585 (64)	153 (52) **	28 (62)	**0.001**
Pack/year	-	10 [15]	9.05 [17.2]	0.460
Smoking cessation, y	-	15 [22]	-	NA
Race, Caucasian, *n* (%)	848 (93)	279 (95)	41 (91)	0.366
BMI, kg∙m^2^	25.6 [6.1]	25.5 [5.7]	24.7 [6.6]	0.178
Atopy, *n* (%)	395 (72)	136 (73)	19 (86)	0.319
*Comorbidities*				
Rhinitis, *n* (%)	570 (64)	194 (67)	24 (56)	0.325
Chronic rhinosinusitis, *n* (%)	242 (29)	96 (35)	11 (26)	0.174
Nasal poliposis, *n* (%)	404 (46)	124 (43)	13 (29)	0.093
Bronchiectasis, *n* (%)	165 (22)	50 (20)	4 (11)	0.341
GERD, *n* (%)	326 (37)	120 (42)	12 (27)	0.126
Atopic dermatitis, *n* (%)	58 (6.5)	14 (4.8)	6 (14)	0.060
Urticaria, *n* (%)	46 (5.2)	16 (5.6)	4 (9)	0.532
Psoriasis, *n* (%)	16 (1.8)	9 (3.2)	0 (0)	0.239
Chronic cardiovascular disease, *n* (%)	189 (25)	84 (32)	10 (26)	0.085
Depression, *n* (%)	41 (5.4)	15 (5.7)	3 (7.5)	0.845
Anxiety, *n* (%)	67 (8.9)	27 (10.5)	4 (10.3)	0.720
Diabetes, *n* (%)	39 (5.1)	16 (6.2)	2 (5.1)	0.804
Osteoporosis, *n* (%)	121 (17.1)	40 (16.6)	2 (5.6)	0.190

Data are shown as numbers of patients (percentage) or medians [interquartile range]. Percentages are calculated for non-missing data. Abbreviations: BMI indicates body mass index; GERD, gastroesophageal reflux disease. * and **: *p* < 0.05 and *p* < 0.001 vs. no smokers group; § and §§: *p* < 0.05 and *p* < 0.001 vs. former smokers group. The bold highlights the statistically significant *p*-value.

**Table 2:** Functional, clinical, and laboratory variables of study population.

	No Smokers	Former Smokers	*p*-Value	Former Smokers	Former Smokers	*p*-Value	Former Smokers	Former Smokers	*p*-Value
	(N = 910)	(N = 293)		(<10 Pack/Years)	(≥10 Pack/Years)		(<15 Years of Smoking Cessation)	(≥15 Years of Smoking Cessation)	
				(N = 124)	(N = 152)		(N = 130)	(N = 134)	
Pre-BD FEV_1_, L	1.98 [1.10]	2.02 [1.01]	0.990	2.06 [1.03]	2 [0.98]	0.845	2.06 [0.96]	1.97 [1.02]	0.066
Pre-BD FEV_1_, % pred.	74 [29.3]	72 [29.7]	0.354	73 [34.5]	71.6 [27]	0.757	72 [29]	74 [33.7]	0.779
Post-BD FEV_1_, L	2.11 [1.07]	2.09 [1.14]	0.615	2.42 [1.29]	2.01 [1.07]	0.638	2.45 [1.39] *	1.90 [1.12]	**0.012**
Post -BD FEV_1_, % pred.	80 [29]	83.4 [38.7]	0.303	83.8 [41]	85 [29.4]	0.782	88.5 [30.5]	82 [43]	0.672
Pre-BD FEV_1_/FVC, %	68.29 [16.63]	67 [16.76]	**0.020**	67.9 [18.2]	66.8 [17.5]	0.580	68.4 [15.6]	66.9 [17.2]	0.931
Post-BD FEV_1_/FVC, %	72.4 [17.61]	66.7 [19.5]	**0.003**	67.7 [13.8]	65.6 [19.9] *	0.410	67.5 [18.8] *	65.8 [18.7] *	0.981
FeNO, ppb	31 [40.5]	32.5 [40.5]	0.920	34 [42]	33 [40]	0.743	30.5 [30.2]	35 [41.7]	0.247
ACT, score	18 [8]	18 [8]	0.809	18 [8]	17 [9]	0.140	17 [9]	18 [8]	0.318
ACQ, score	2.42 [2.60]	2.33 [2.61]	0.892	2.20 [2.39]	2.42 [2.72]	0.111	2.37 [2.86]	2.24 [2.61]	0.202
Blood Eosinophils, cell/ul	0.35 [0.57]	0.41 [0.61]	0.130	0.38 [0.62]	0.42 [0.60]	0.572	0.41 [0.59]	0.41 [0.66]	0.910
Blood Eosinophils, %	4.04 [6.6]	5.25 [7.4]	0.137	4.5 [8.5]	6 [7.1]	0.753	5 [7.4]	6.2 [8.4]	0.594
Serum IgE, U/ul	195 [372]	240.5 [576]	0.165	211.5 [475.9]	277 [656]	0.240	257.5 [639.3]	224 [440.4]	0.276
Blood lymphocytes, cell/ul	2.14 [0.94]	2.11 [1.04]	0.970	2.14 [0.89]	2.09 [1.23]	0.948	2.34 [1.24] *	2 [0.85]	**0.004**
Blood lymphocytes, %	30 [11.5]	30 [10.9]	0.352	30.4 [11.5]	29 [9.62]	0.612	30.2 [10.8]	28.5 [9.4]	0.202
Blood neutrophils, cell/ul	4.09 [2.21]	4.10 [2.29]	0.602	4.1 [2.17]	4.10 [2.31]	0.982	4.24 [2.43]	3.9 [1.89]	**0.039**
Blood neutrophils, %	55.3 [13.50]	54.9 [12.35]	0.816	55.1 [14.2]	54.9 [11.5]	0.591	53.5 [11.4]	54.8 [13.7]	0.328
Days lost at work, n/y	0 [7]	0 [6]	0.782	0 [5]	0 [8]	0.443	0 [10]	0 [4]	0.180
Severe exacerbations, n/y	2 [3]	2 [3]	0.934	2 [3]	2 [4]	0.689	2 [4]	2 [3]	0.116
ED admissions, n/y	0 [0]	0 [0]	0.440	0 [0]	0 [0]	0.413	0 [0]	0 [0]	0.444
Hospitalizations, n/y	0 [0]	0 [0]	0.926	0 [0]	0 [0]	0.654	0 [0]	0 [0]	0.081

Data are shown as numbers of patients (percentage) or medians [interquartile range]. Percentages are calculated for non-missing data. Abbreviations: BD indicates bronchodilator; FEV_1_, forced expiratory volume in the 1st second; FVC, forced vital capacity; FeNO, fractional exhaled nitric oxide; ACT, asthma control test; ACQ, asthma control questionnaire; IgE, immunoglobulin E; ED, emergency department. * *p* < 0.05 vs. no smokers group. The bold highlights the statistically significant *p*-value.

**Table 3:** Study population clustered by blood eosinophilia.

	Eosinophils < 0.30	Eosinophils ≥ 0.30	*p*-Value
	(*N* = 396)	(*N* = 532)	
No smokers/Former smokers, *n* (%)	310 (78)/86 (22)	389 (73)/143 (27)	0.071
Pre-BD FEV_1_, L	1.98 [1.02]	1.98 [1.08]	0.763
Pre-BD FEV_1_, % pred.	77.1 [30.6]	71.6 [28.9]	**0.024**
Post-BD FEV_1_, L	2.11 [1.14]	2.07 [1.09]	0.965
Post -BD FEV_1_, % pred.	83 [30.7]	77.6 [32]	**0.031**
Pre-BD FEV_1_/FVC, %	69.7 [15.9]	67.5 [15.5]	0.081
Post-BD FEV_1_/FVC, %	72.2 [17.8]	69.7 [16]	0.272
FeNO, ppb	24 [35.7]	39 [43]	**<0.001**
ACT, score	19 [7]	17 [8]	**<0.001**
ACQ, score	2.18 [2.33]	2.85 [2.40]	**<0.001**
Days lost at work, *n*/y	0 [6]	0 [10]	**0.021**
Severe exacerbations, *n*/y	2 [3]	2 [4]	**0.013**
ED admissions, *n*/y	0 [0]	0 [0]	0.083
Hospitalizations, *n*/y	0 [0]	0 [0]	0.746

The bold highlights the statistically significant *p*-value.

## Data Availability

The full dataset supporting the reported results is available upon request to the corresponding author.
